# Triplet Therapy in Melanoma — Combined BRAF/MEK Inhibitors and Anti-PD-(L)1 Antibodies

**DOI:** 10.1007/s11912-022-01243-x

**Published:** 2022-04-02

**Authors:** Julia R. Dixon-Douglas, Riyaben P. Patel, Pretashini M. Somasundram, Grant A. McArthur

**Affiliations:** 1 Department of Medical Oncology, Peter MacCallum Cancer Centre, Melbourne, VIC Australia; 2 Cancer Research Division, Peter MacCallum Cancer Centre, Melbourne, VIC Australia; 3 Faculty of Medicine, Dentistry and Health Sciences, University of Melbourne, Parkville, VIC Australia; 4 Department of Oncology, Sir Peter MacCallum, University of Melbourne, Parkville, VIC Australia

**Keywords:** Advanced melanoma, BRAF mutant, BRAF/MEK inhibitors, Immune checkpoint inhibitors, Immunotherapy

## Abstract

**Purpose of Review:**

We provide an updated review of clinical trials evaluating the combination of BRAF/MEK inhibitors with anti-PD-(L)1 therapy (triplet therapy) for patients with advanced BRAF-mutant melanoma, accompanied by a summary of the biological evidence supporting this combination.

**Recent Findings:**

Resistance to BRAF/MEK inhibition and comparatively low response rates to immune checkpoint inhibitors remain clinical challenges in the treatment of melanoma. Preclinical data demonstrates that targeted therapy is immune-modulatory and synergises with immune checkpoint inhibition. Several randomised controlled trials have evaluated the combination of targeted therapy with immune checkpoint inhibition.

**Summary:**

Triplet therapy has shown improvements in progression-free survival and durability of response compared to BRAF/MEK inhibition alone; however, questions remain regarding the best clinical scenario for implementation of this regimen in the era of front-line immunotherapy.

## Introduction

Current strategies for the treatment of advanced BRAF-mutant melanoma include targeted therapies in the form of BRAF/MEK inhibitors (BRAF/MEKi) and immune checkpoint inhibitors (CPI), including antibodies to programmed cell death-1 (anti-PD-1), programmed cell death ligand-1 (anti-PD-L1) and cytotoxic T lymphocyte antigen (anti-CTLA4). Targeted therapies are characterised by the induction of rapid but often short-lived responses whilst CPI have led to unprecedented long-term disease control, albeit with lower response rates. Preclinical models and translational data demonstrate that BRAF/MEKi have an immunomodulatory effect on the tumour microenvironment (TME), further strengthening the biological rationale of combining the complimentary clinical profiles of BRAF/MEKi and CPI. In recent years, the combination of BRAF/MEKi and CPI has been evaluated in several randomised controlled trials (RCTs). Here, we provide an updated review of available clinical data and an accompanying summary of the biological basis of combined BRAF/MEKi and CPI, so-called triplet therapy.

### Clinical Rationale

BRAFV600 mutations occur in approximately 35–50% of cutaneous melanoma and result in constitutive activation of the MAPK pathway [1]. BRAF-mutant melanomas are sensitive to blockade of this pathway with BRAF inhibitors which reduce tumour cell survival and proliferation. Despite these clinical gains, the utility of BRAFi monotherapy has been limited by both acquired resistance and the development of cutaneous toxicity secondary to paradoxical MAPK pathway upregulation. As such, the combination of a MEKi with a BRAFi has been developed to reduce both the development of resistance and clinical toxicity [2, 3]. BRAF/MEKi combinations, including dabrafenib/trametinib, vemurafenib/cobimetinib and encorafenib/binimetinib, are associated with high objective response rates (ORRs), in the order of 70%, for advanced BRAF-mutant melanoma [4–6]. However, resistance to targeted therapies develops in the vast majority of patients, reflected in a relatively short median progression-free survival (PFS) of 10–15 months and 5-year overall survival rates of 34–37% [4–7]. In contrast, immune checkpoint inhibitors have achieved an unprecedented 5-year overall survival rate of 52% with the combination of ipilimumab and nivolumab [8]. Notably, an even higher 5-year overall survival rate of 60% is seen in the BRAF-mutant subgroup [8, 9]. Additionally, retrospective data suggests that the efficacy of CPI is likely to be reduced in patients who have acquired resistance to BRAF/MEKi [10–12]. Whether the high response rates of BRAF/MEKi can be combined with the durability of CPI in the front-line setting is therefore of significant clinical interest.

### Biological Rationale

In addition to their direct effects on tumour cells through blockade of the MAPK pathway, BRAF/MEKi have also been shown to modulate the TME [13]. Preclinical studies using mouse melanoma models and patient samples have revealed that BRAF/MEKi initially induce an immune-stimulatory TME, which contributes to their therapeutic activity [14–16]. This immune-stimulatory tumour microenvironment (TME) is characterised by reduced infiltrates of immunosuppressive cell types, including T-regulatory cells [13] and myeloid-derived suppressor cells [14], and decreased levels of immunosuppressive cytokines including IL-10 and IL-8 [17, 18]. BRAF/MEKi also promote the recruitment and/or expansion of cytotoxic CD8 + T-cells [19], increase T-cell killing [20] and enhance T-cell recognition of melanoma cells by promoting HLA class I [17] and antigen expression on tumour cells [17].

**Fig. 1:**
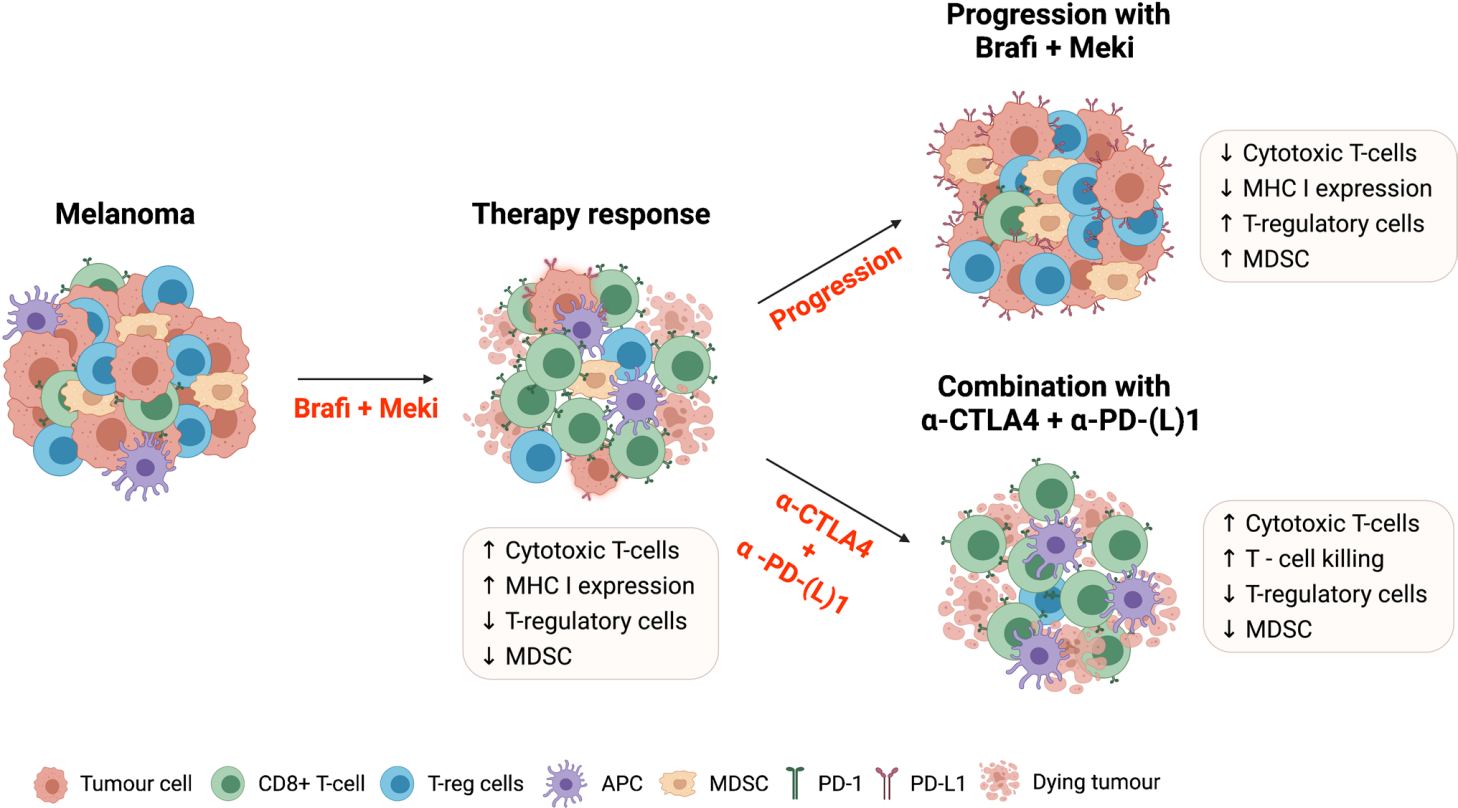
Immune checkpoint blockade enhances the pro immunogenic effect of BRAFi + MEKi on the melanoma tumour microenvironment. Melanoma is an immunogenic tumour, characterised by a high neoantigen load and immune cell infiltrates including immunostimulatory cytotoxic T-cells (CTL) and immunosuppressive T-regulatory (T-reg) and myeloid-derived suppressor cells (MDSC). Upon initial treatment with BRAFi + MEKi, an immunogenic tumour microenvironment (TME) is created characterised by increased CTLs, reduced T-reg and MDSCs and increased recognition and targeting of melanoma cells. However, upon progression on targeted therapy, an immunosuppressive TME develops, ultimately enabling melanoma escape. This is characterised by increased T-reg and MDSCs, reduced CTLs and reduced melanoma antigen presentation, recognition and elimination by T-cells. Melanoma cells also increase expression of programmed death ligand-1 (PD-L1), which ligates the programmed cell death protein-1 (PD-1) checkpoint on the surface of T-cells, further dampening their cytotoxic activities. Through combining BRAFi + MEKi with immune checkpoint blockade, the development of an immunosuppressive TME is abrogated by increasing CTL mediated killing of melanoma cells and reducing T-reg and MDSC infiltrates

In contrast, an immunosuppressive TME at the time of progression on BRAF/MEKi therapy has been observed in both preclinical and clinical studies. Resistance to BRAF/MEKi is associated with upregulation of PD-L1 on both melanoma cells and immune cells [21, 22], a reduction in the ratio of CD8 + T-cell to T-regulatory cells [14, 15, 17] and an increase in T-cell exhaustion markers such as TIM-3 and PD-1 [17]. A reduction in granzyme B and perforin has also been noted, indicating a reduction in T effector cell function [23]. Long-term BRAF/MEKi treatment has been shown to impair antigen-presenting mechanisms leading to a further reduction in cytotoxic CD8 + T-cell activity [24, 25]. A recent study suggests that this immune-evasive state is driven by reactivation of the MAPK pathway in BRAF/MEKi resistant tumours, which also underpins resistance to subsequent immunotherapy, so-called cross-resistance [23]. A biological rationale has thus emerged to support combining targeted therapy and immune checkpoint blockade: to harness the early immune-stimulatory effects of BRAF/MEKi and to abrogate the emergence of the immunosuppressive environment that drives treatment resistance (Fig. 1). This could translate to clinical benefits including preservation of high response rates with increased durability of response, ultimately increasing patient survival.

## Preclinical Data

To test the hypothesis of improved anti-tumour immunity with triplet therapy, preclinical studies have examined co-administration of BRAF/MEKi and anti-PD-1 or anti-PD-L1 antibodies in melanoma mouse models. In a syngeneic mouse model of SM1 BRAFV600E mutant melanoma, triplet therapy demonstrated heightened anti-tumour activity compared with either anti-PD-1 monotherapy or anti-PD-1 in combination with either BRAFi or MEKi alone [26, 27]. This was further enhanced by the addition of immune-stimulatory antibody anti-CD137 [27]. Similar synergy was also demonstrated in a BRAFV600E/Pten^(−/−)^ melanoma mouse model demonstrating prolonged survival and slowed tumour growth in mice treated with combined BRAFi and anti-PD-1 or anti-PD-L1, whereas anti-PD-(L)1 monotherapy failed to induce response [14]. In both of these models, combination therapy was associated with a greater influx of cytotoxic CD8 + T-cells and a higher ratio of CD8 + to T-regulatory cells than either treatment alone [14, 26]. Higher levels of granzyme B-positive T-cells, INF-gamma-positive and TNF-positive CD8 + T-cells were observed, indicative of increased T-cell functionality and cytotoxicity [14, 26]. In another study, even short-term dual inhibition of BRAF/MEK, when combined with anti-PD-1, was sufficient to enhance tumour immune infiltration and overall improved tumour control in a CD8 + T-cell dependent manner [28]. Overall, preclinical data from melanoma mouse models provide robust foundational evidence to support synergistic anti-tumour activity mediated through enhanced immunogenicity of the TME.

## Translational Data from Clinical Studies

Observations from early phase clinical trials have assisted in refining this biological hypothesis and have informed the design of current clinical trials. Translational studies have sought to characterise immune cell infiltrates of serial tumour biopsies collected from melanoma patients on treatment and at the time of progression. One such study of patients treated with a BRAF inhibitor showed an early increase in CD8 + T-cell infiltrates after 3–15 days on treatment, which correlated with a decrease in tumour size [15]. This was followed by a decrease in T-cell infiltrates at the time of progression [15]. Tumour biopsies from a phase I clinical trial, where a patient was treated with 4 weeks of vemurafenib followed by 4 cycles of anti-CTLA4 antibody ipilimumab [29], demonstrated an influx of CD8 + T-cells and increased CD8 + to T-regulatory cell ratio at day 8 [14]. At day 35, these changes had regressed and were virtually absent, although recurred and persisted (> 70 days) following subsequent treatment with ipilimumab. These data suggest that maximal synergistic anti-tumour immunity is likely achieved early after initiation of BRAF/MEK inhibition.

### Clinical Trials

Initial phase I clinical trials of triplet therapy explored the safety and feasibility of combining BRAF/MEKi with the anti-CTLA4 antibody ipilimumab. This combination resulted in prohibitive gastrointestinal toxicity. Dose-limiting hepatotoxicity was observed in 6 of 10 patients treated with the combination of vemurafenib and ipilimumab [29] and colitis with intestinal perforation was reported in 2 of 7 patients receiving a combination of dabrafenib, trametinib and ipilimumab [30]. Subsequent efforts have focused on combining BRAF/MEK inhibitors with anti-PD-1 and anti-PD-L1 antibodies, resulting in a more favourable toxicity profile and response rates of 64–85% across trials [31–33]. Manageable toxicity for the combination of dabrafenib and trametinib with either pembrolizumab or spartalizumab at standard monotherapy doses has been established [31, 34]. In a phase I trial, concurrent initiation of vemurafenib and atezolizumab resulted in grades 3–4 toxicity in 3 of 3 patients [32]. Subsequently, the combination of vemurafenib, atezolizumab and cobimetinib was shown to be tolerable following a 28-day run-in of vemurafenib and cobimetinib [32]. Most recently, results of the phase I IMMU-TARGET trial have demonstrated the safety of combination encorafenib, binimetinib and pembrolizumab at standard doses with an ORR response rate of 64% [33]. Following on from these early efficacy signals, several RCTs have been conducted to confirm the efficacy of combined BRAF/MEK inhibitors with CPI compared to BRAF/MEK inhibitors alone in the first-line setting.

#### Clinical Trial Design and Population

Three RCTs have evaluated the combination of BRAF/MEKi with either anti-PD-1 or anti-PD-L1 antibodies in patients with unresectable or metastatic melanoma. A fourth RCT is ongoing (STARBOARD, NCT04657991) comparing encorafenib, binimetinib and pembrolizumab to pembrolizumab monotherapy is yet to be reported.

Part 3 of the KEYNOTE-022 phase I/II clinical trial randomised 120 patients with unresectable stage III or IV BRAFV600E or V600K mutant melanoma to receive dabrafenib 150 mg BID and trametinib 2 mg daily in combination with either pembrolizumab 200mg 3-weekly or placebo [35]. The phase III IMspire150 trial, otherwise known as TRILOGY, compared the combination of vemurafenib, cobimetinib and atezolizumab to vemurafenib, cobimetinib and placebo in 514 patients with unresectable stage IIIC or IV melanoma [36]. All patients underwent a 28-day run-in of vemurafenib 960 mg BID and cobimetinib 60 mg daily (days 1 to 21); after which, the atezolizumab group received a lower dose of vemurafenib (720 mg BID + vemurafenib placebo) consistent with the recommended schedule from the phase I trial [32]. From cycle 2 onwards, atezolizumab 840mg 2-weekly or placebo was added to these respective dosing schedules. Finally, part 3 of the COMBI-i trial randomised 532 patients with unresectable stage IIIC or IV melanoma to receive dabrafenib 150 mg BID and trametinib 2 mg daily with either spartalizumab 400 mg 4-weekly or placebo [37]. Patients with unresectable stage III disease comprised a small proportion (5–6%) of the overall population in each of these studies. There were some differences in the recognised adverse prognostic factors between trial populations, including elevated lactate dehydrogenase (LDH) levels. Patients with advanced stage of disease with visceral metastases (stage M1c) were similarly represented in all three trials, although a higher proportion of patients with stage M1c and high LDH was noted in the pembrolizumab arm of KEYNOTE-022 (Table 1).

**Table 1:** Key patient characteristics

	KEYNOTE-022 *N* = 120		IMspire150 *N* = 514		COMBI-i *N* = 532	
	D + T + Pem	D + T + Pbo	V + C + Atezo	V + C + Pbo	D + T + Sparta	D + T + Pbo
*Age, years*	54	58	54	53.5	56	55
*ECOG*						
* 0*	78	73	76	77	73	74
* 1*	22	27	24	22	25	25
*2*	-	-	-	-	2	1
*LDH at baseline* > *ULN (%)*	45	43	33	33	39	40
*Melanoma staging (%)*						
* IIIB*	2	2	-	-	-	-
* IIIC*	0	3	5	6	6	6
* IVa*	3	17	16	14	11	16
* IVb*	13	15	22	16	21	14
* IVc*	82	63	57	63	62	65

Data for age is in median, all other data is %. *D* + *T*, dabrafenib and trametinib; *V* + *C*, vemurafenib and cobimetinib; *Pem.*, pembrolizumab; *Atezo.*, atezolizumab; *Sparta.*, spartalizumab; *Pbo*, placebo; *N*, number randomised; *ECOG*, Eastern Co-operative Oncology Group performance status; *LDH*, lactate dehydrogenase level; *ULN*, upper limit of normal. Melanoma staging is according to American Joint Committee on Cancer Melanoma Staging, 7th Edition

#### Clinical Efficacy

Each of the three RCTs reported to date have yielded numerically similar results with respect to their uniform primary endpoint of investigator-assessed PFS with results of 16.9, 15.1 and 16.2 months in the triplet arms compared to 10.7, 10.6 and 12.0 months in the placebo arms of KEYNOTE-022, IMspire150 and COMBI-i, respectively. However, only the IMspire150 trial demonstrated a statistically significant improvement in PFS with a hazard ratio (HR) of 0.78 and a *p*-value of 0.0249. KEYNOTE-022 failed to reach significance at the pre-specified analysis but notably achieved a HR of 0.53 in the 36-month post hoc analysis, suggesting longer follow up is required to demonstrate the true benefit of triplet therapy compared to doublet therapy. The control arm in COMBI-i performed somewhat better than expected (Table 2), which may have limited the ability to show a statistically significant benefit for the triplet regimen (HR for PFS 0.82, one-sided *p*-value 0.042, threshold for significance *p*-value < 0.025).

**Table 2:** Primary and secondary efficacy endpoints

	PFS (months)	ORR (%)	DOR (months)	OS at 24 months (%)
KEYNOTE-022				
* Dabrafenib* + *trametinib* + *pembrolizumab* vs * Dabrafenib* + *trametinib* + *placebo*	16.9 10.7 (HR 0.53)	63.3 71.7	25.1 12.1	63 52
IMspire150				
* Vemurafenib* + *cobimetinib* + *atezolizumab* vs * Vemurafenib* + *cobimetinib* + *placebo*	15.1 10.6 (HR 0.78, *p* = 0.0249)	66.3 65.0	21.0 12.6	60.4 53.1
COMBI-i				
* Dabrafenib* + *trametinib* + *spartalizumab* vs * Dabrafenib* + *trametinib* + *placebo*	16.2 12.0 (HR 0.82, *p* = 0.042)	68.5 64.2	NR 20.7	68 62

*PFS*, median progression-free survival (months); *HR*, hazard ratio; *ORR*, objective response rate (%); *DOR*, duration of response (months); *OS*, overall survival (%)

ORR, duration of response (DOR) and overall survival (OS) were key secondary endpoints across the three trials. Although the pembrolizumab arm of KEYNOTE-022 surprisingly showed a lower ORR (63%) compared to the control arm (72%), this finding was not replicated in either of the phase III trials, and may have been due to the higher proportion of stage M1c patients in the triplet arm of this study.

Importantly, the duration of response was demonstrably longer with triplet therapy across all three studies (Table 2), supporting the hypothesis that the addition of CPI extends the longevity of response to BRAF/MEKi. There was a trend to improved overall survival in all three trials, although this data remains immature. In KEYNOTE-022, the median overall survival has not yet been reached in the triplet arm, compared to 26.3 months (HR 0.64) in the placebo arm, and has not yet been reached in either arm of the IMspire150 and COMBI-i trials.

Subgroup analyses demonstrated a consistent trend towards improved PFS with triplet therapy across patient age, performance status, LDH level and disease stage across all three trials. The COMBI-i trial has reported a biomarker analysis evaluating PFS according to tumour mutational burden (TMB). Two hundred thirty-seven patients had low TMB (defined as < 10 mutations per megabase) and 177 patients had high TMB (10 or more mutations per megabase). A greater numerical improvement in PFS (23.9 m vs. 11.8 m, HR 0.703) was seen in the high TMB group but this did not meet statistical significance [37].

#### Safety

Improvements in durability and efficacy are offset to an extent by an increase in toxicity observed in the combination arms of all three trials. Increased rates of fever, rash, diarrhoea and liver transaminase elevation were observed with triplet regimens. Substantially higher rates of treatment-related grades 3–5 toxicity were seen in the triplet arm compared to the doublet arm of KEYNOTE-022 and COMBI-i (58.3% vs. 25% and 54.7% vs. 33.3%, respectively). There was one treatment-related death (due to pneumonitis) in KEYNOTE-022. Although the reported rates of treatment-related grades 3–4 toxicity in IMspire150 exceed 70%, toxicity profiles between the combination and placebo arms were comparable (79% and 73%, respectively), and largely driven by asymptomatic and reversible laboratory abnormalities, including elevated creatine phosphokinase, and the rate of treatment discontinuation in this trial was low (13% and 16% in the combination and placebo arms, respectively). Similarly, discontinuation for treatment-related adverse events in COMBI-i was low at 12.4% (spartalizumab arm) compared to 8% (control arm). In contrast, toxicity of the triplet regimen in KEYNOTE-022 led to discontinuation in 47% of patients (predominately due to hepatitis but notably also several cases of grade 3 pneumonitis), compared to 20% in the control arm.

## Clinical Utility and Limitations

Whilst only one of the three randomised controlled trials to date has met its primary endpoint in terms of a statistical improvement in PFS, the strikingly consistent numerical increase in PFS and duration of response in the triplet arms does support a role for the addition of anti-PD-1 to BRAF/MEK inhibitors. Where these triplet regimens will sit in the clinical armamentarium has been challenged by the evolution of combination ipilimumab and nivolumab (IPI + NIVO) as the current front-line standard of care for unresectable melanoma [8, 9, 38]. The major limitation of the triplet therapies is the lack of IPI + NIVO comparator arm, which was not standard of care at the time of trial design. An additional argument for the use of upfront IPI + NIVO in BRAF-mutant melanoma is the particular overall survival benefit seen with this regimen in this specific patient sub-population [8]. Overall survival data for the triplet therapies remains immature, and is likely to be mitigated to an extent by post-study therapy, which may include combination immune checkpoint inhibition for some patients.

There may be a role for triplet therapies in patients for whom IPI + NIVO is contraindicated or undesirable given the high rates of grades 3–4 immune-mediated toxicity associated with IPI + NIVO. Whilst the toxicity of triplet therapy is not insubstantial, the profile is arguably more tolerable than that of IPI + NIVO, with the exception of the KEYNOTE-022 regimen [9, 35, 36]. Another group of interest is the good-prognosis low-volume disease, normal LDH group, who may not derive major overall survival benefit from the addition of ipilimumab to single-agent nivolumab [8]. It is possible that these patients stand to gain incremental benefit from the addition of upfront BRAF/MEK inhibitors to single-agent anti-PD-1. However, there is no data demonstrating the superiority of the triplet regimen over single-agent anti-PD-1. Furthermore, the novel immune checkpoint combination of anti-LAG3 antibody relatlimab with nivolumab has recently demonstrated promising progression-free survival advantage over single-agent anti-PD-1 with minimal added toxicity [39], adding an additional immunotherapy option for this group of patients.

## Future Directions

These trials demonstrate that the combination of BRAF/MEKi/CPI is a feasible and active treatment option. In the context of an evolving treatment landscape with multiple immune checkpoint combinations now available, the future of the triplet regimen will depend on identifying the ideal clinical scenario for implementation, which may be in well-defined patient sub-populations or beyond the front-line metastatic setting.

### Anti-PD-1 Refractory Melanoma

The TRIdent study is a single-arm phase II study of dabrafenib, trametinib and nivolumab that notably included anti-PD-1 refractory patients and patients with brain metastases who were BRAF-mutant and BRAF/MEKi-naïve. An objective response rate of 88% (14 of 16 patients) was achieved in the anti-PD-1 refractory group, and an intracranial response rate of 57% was seen in patients with brain metastases [40].

### Adjuvant/Neoadjuvant Setting

Triplet therapies may have a future role in the adjuvant or neoadjuvant setting. Both BRAF/MEKi and anti-PD-1 have demonstrated efficacy in this area [41–43]. The single-arm phase II trial Neo-VC (NCT02303951) investigating the combination of vemurafenib, cobimetinib and atezolizumab as a neoadjuvant strategy for borderline resectable stage IIIC/IV melanoma closed early due to poor recruitment. The ongoing Neo Trio study (NCT02858921) is investigating several combinatorial schedules: schedule 1 consists of 6 weeks of combined neoadjuvant dabrafenib/trametinib and pembrolizumab, schedule 2 consists of a sequential approach of 2 weeks of neoadjuvant dabrafenib/trametinib, followed by pembrolizumab, and schedule 3 consists of pembrolizumab alone pre-operatively; all arms receive adjuvant pembrolizumab post-operatively.

### Sequencing

The observation from preclinical and translational data that the effect of BRAF and MEK inhibitors on the tumour microenvironment is dynamic and temporally dependent raises the possibility that sequential administration of BRAF/MEKi and CPI could optimise anti-tumour immunogenicity. As yet, there is no completed randomised, prospective data to inform the optimum sequence of BRAF/MEK inhibitors and CPI therapies in patients with BRAF-mutant melanoma. The recently presented interim results from the DREAMseq trial indicate improved 2-year PFS and OS rates with upfront IPI + NIVO followed by dabrafenib and trametinib on progression, compared to the reverse sequence [44]. However, these survival curves cross at approximately 6 and 10 months respectively with a proportion of early deaths in the upfront IPI + NIVO arm, reinforcing the need to induce rapid response with targeted therapy in certain patients. The SECOMBIT study has a similar design of IPI + NIVO followed by encorafenib and binimetinib on progression or vice versa, and includes a third arm comprising an 8-week run-in period of encorafenib and binimetinib, followed by IPI + NIVO until progression, with subsequent encorafenib and binimetinib rechallenge. This strategy theoretically incorporates priming of the TME with targeted agents, followed by a switch to immunotherapy before resistance is established. The SECOMBIT study has reported preliminary results; however, data remain immature for the assessment on the primary endpoint of overall survival [45]. Several other clinical trials evaluating sequencing of immunotherapy and targeted therapy are ongoing, including ImmunoCobiVem (NCT02902029) and EORTC-1612-MG (NCT03235245).

## Conclusion

Combining the clinically diametric profiles of BRAF/MEK inhibitors and CPI is supported by strong biological rationale. Namely, an immune-stimulatory effect of BRAF/MEKi, implication of immune-exhaustion in acquired resistance to BRAF/MEKi and synergy between BRAF/MEKi and CPI in preclinical models have all been observed. Three randomised clinical trials evaluating combination BRAF/MEKi and CPI in the first-line setting demonstrate strikingly similar numerical and clinically significant improvements in progression-free survival and durability of response compared to targeted therapy alone. The clinical application of triplet therapies in the first-line metastatic setting has been limited by a lack of comparison to either single-agent anti-PD-1 or IPI + NIVO, which have since emerged as standards of care for both BRAF-mutant and wild-type melanoma. Nonetheless, both preclinical and clinical data to support the triplet regimen is compelling. Investigation of how best to apply this synergy clinically is ongoing and includes evaluation in the (neo)adjuvant setting as well as investigation of sequential and alternating therapies. Such research must include translational objectives and patient biospecimens to deepen our understanding of the interaction between BRAF/MEKi, CPI, tumour characteristics and the immune microenvironment to direct patient selection or to unveil novel therapeutic strategies.
